# Prevalence and distribution of human *Plasmodium* infection in Pakistan

**DOI:** 10.1186/1475-2875-12-297

**Published:** 2013-08-28

**Authors:** Aamer A Khattak, Meera Venkatesan, Muhammad F Nadeem, Humayoon S Satti, Adnan Yaqoob, Kathy Strauss, Lubna Khatoon, Salman A Malik, Christopher V Plowe

**Affiliations:** 1 Department of Biochemistry, Faculty of Biological Sciences, Quaid-i-Azam University, Islamabad, Pakistan; 2 Howard Hughes Medical Institute/Center for Vaccine Development, University of Maryland School of Medicine, Baltimore, MD, USA; 3 WorldWide Antimalarial Resistance Network Molecular Module, University of Maryland School of Medicine, Baltimore, MD, USA; 4 King Edward Medical University, Lahore, Pakistan; 5 Department of Biochemistry and Molecular Biology, University of Gujrat, Gijrat, Pakistan

**Keywords:** *Plasmodium falciparum*, *Plasmodium vivax*, Malaria, Pakistan

## Abstract

**Background:**

Both *Plasmodium vivax* and *Plasmodium falciparum* are prevalent in Pakistan, yet up-to-date data on the epidemiology of malaria in Pakistan are not available. This study was undertaken to determine the current prevalence and distribution of *Plasmodium* species across the country.

**Methods:**

A malariometric population survey was conducted in 2011 using blood samples collected from 801 febrile patients of all ages in four provinces and the capital city of Islamabad. Microscopically confirmed *Plasmodium*-positive blood samples were reconfirmed by polymerase chain reaction (PCR). Confirmed parasite-positive samples were subjected to species-specific PCR capable of detecting four species of human malaria.

**Results:**

Of the 707 PCR-positive samples, 128 (18%) were *P. falciparum*, 536 (76%) were *P. vivax*, and 43 (6%) were mixed *P. falciparum* and *P. vivax*. Ninety-four microscopy-positive samples were PCR-negative, and *Plasmodium malariae* and *Plasmodium ovale* were not detected. Prevalence of *P. vivax* ranged from 2.4% in Punjab Province to 10.8% in Sindh Province and prevalence of *P. falciparum* ranged from 0.1% in Islamabad to 3.8% in Balochistan.

**Conclusions:**

*Plasmodium* infections in Pakistan are largely attributed to *P. vivax* but *P. falciparum* and mixed species infections are also prevalent. In addition, regional variation in the prevalence and species composition of malaria is high.

## Background

Ninety-five million of Pakistan's 161 million people, roughly 60% of Pakistan’s population, live in malaria-endemic regions [1,2]. After eradication efforts in the 1960s, malaria surged back to an epidemic level in the 1970s. In recent years, an uptick in malaria can be partially attributed to floods that affected approximately 20 million people in over 60 districts [2]. Despite a well-established malaria control programme, 500,000 malaria infections and 50,000 malaria-attributable deaths occur each year in Pakistan [3], with approximately 37% of cases estimated to occur in regions along the borders with Afghanistan and Iran [4].

Pakistan’s climate ranges from tropical to temperate with dry conditions along the southern coast [5], and elevation ranges from sea level to nearly 9,000 meters [6]. *Plasmodium vivax* (responsible for approximately 64% of infections) and *Plasmodium falciparum* (causing 36% of infections) are the two prevalent *Plasmodium* species in Pakistan [7], and malaria is primarily found in the provinces of Khyber Pakhtunkhwa, Balochistan, Sindh and the Federally Administered Tribal Areas [8]. Malaria transmission is considered to be unstable, with major *P. vivax* transmission peaking from June to September and again in April to June, when relapses of infections acquired the previous season are observed [9]. The major transmission period for *P. falciparum* in Pakistan is between August and December [9].

Although the majority of malaria cases in Pakistan is caused by *P. vivax*, trends in the past few decades have indicated that *P. falciparum* infection is on the rise. The World Health Organization (WHO) reported that in Pakistan the proportion of malaria infections attributed to *P. falciparum* rose from 34% in 1987 to 54% in 1990 [9,10]. The frequency of *P. falciparum* among microscopy-positive cases rose from 45% in 1995 to 68% in 2006 in the city of Quetta in Balochistan province and in Jhangara city of Sindh province [11,12]. In 2010, 73,857 (31%) of 240,591 total reported malaria cases in Pakistan were *P. falciparum*[13].

The rise of *P. falciparum* in parts of Pakistan may be partially attributable to failed treatment of chloroquine-resistant infections [14]. Chloroquine resistance in *P. falciparum* was reported for the first time from Pakistan in 1984 and later confirmed as widespread [13,15-17]. A recent study found that 90% of *P. falciparum* samples collected in the provinces of Balochistan and Sindh carried the *pfcrt* 76 T allele responsible for conferring chloroquine resistance [18]. Although chloroquine is recommended only for treatment of *P. vivax*, *P. falciparum* infections are often treated with chloroquine, as presumptive diagnosis or empiric treatment is usually based on clinical symptoms in resource-limited countries like Pakistan [19].

The heavy influx and continued presence of refugees from Afghanistan, where *P. falciparum* is more prevalent, may also contribute to the increase of *P. falciparum* in Pakistan [20-22] potentiating clinical trials of falciparum malaria in the refugee populations in regions such as Khyber Pakhtunkhwa province [23]. Studies have indicated that refugees have low immunity and are more susceptible to malaria in Pakistan than are permanent residents [10,24,25]. These factors may lead to increased disease and transmission in the refugee population. Monitoring the distribution and burden of *Plasmodium* species in Pakistan as changes occur in both human and parasite populations is necessary to ensure appropriate treatment, particularly in situations where diagnosis by microscopy or species-specific rapid diagnostic tests is not available.

**Figure 1:**
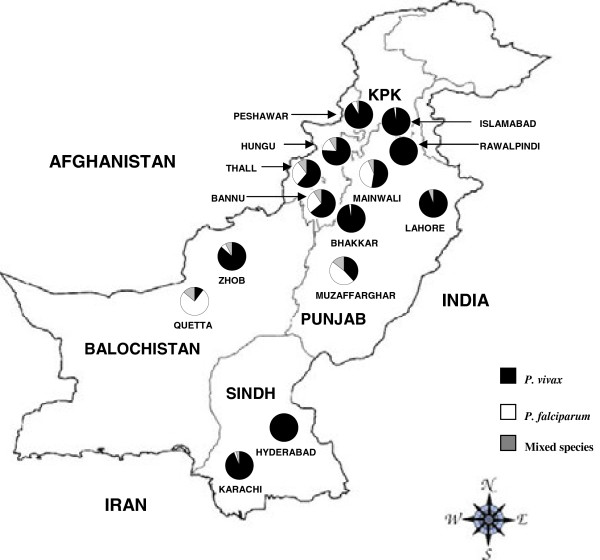
**Sample sites and distribution of *Plasmodium *species across Pakistan, 2011.** KPK: Khyber Pakhtunkhwa province.

A handful of malaria epidemiological studies have been conducted in parts of Pakistan, but the assessment of the malaria burden on a national scale has been hindered by the lack of data from many areas [26]. One report of malariometric surveys conducted from 2005 to 2009 found both spatial and temporal variation in malaria endemicity. Malaria prevalence was highest in the Federally Administered Tribal Areas, followed by Balochistan and Khyber Pakhtunkhwa Provinces [4] (Figure 1), and over the five-year period malaria incidence was highest in 2009 [4]. The aim of this study was to provide a current report on malaria in Pakistan. Surveys were conducted in 2011 in 14 sites across four provinces as well as in the capital city of Islamabad to determine the prevalence of malaria infection among suspected cases and to map the distribution of *Plasmodium* species across the country.

## Methods

Government and private hospitals in 25 cities from four provinces with the highest burden of malaria representing all four provinces (Khyber Pakhtunkhwa province, Sindh province, Balochistan province and Punjab province) and a hospital in the capital city, Islamabad, were invited to participate in the molecular survey. Although the Federally Administered Tribal Areas have among the highest burdens of malaria in the country, they were excluded from this study because political instability and violence [27] make it difficult to establish sample collection. Of the facilities that were contacted, hospitals in 14 cities shared samples and were included in this study: Islamabad (Capital), Peshawar, Thall, Bannu and Hangu (Khyber Pakhtunkhwa province), Karachi and Hyderabad (Sindh province), Zhob and Quetta (Balochistan province), Rawalpindi, Bhakhar, Mainwali, Lahore and Muzaffargarhar (Punjab province) (Figure 1). Samples were collected during the peak malaria transmission season, between April and October 2011, at all sites. The study included patients of all age groups with malarial signs and symptoms (fever, chills, headache, sweats, fatigue, nausea and vomiting). The study was approved by the Institutional Review Board of Quaid-i-Azam University, Pakistan.

For patients giving informed consent, 3 ml of intravenous blood was drawn into an EDTA tube by venepuncture and limited demographic information (patient age and gender) was recorded. Thick and thin films were stained with 10% Giemsa solution and examined at 100× under oil immersion. Smears were examined by a laboratory technician or technologist trained in malaria diagnosis according to WHO guidelines [28]. About 50 μl of each blood sample from 801 malaria microscopy-positive and 30 smear-negative samples was also applied to Whatman 3MM filter paper. Blood spots were air-dried overnight and stored in individual plastic bags with desiccant at room temperature. Remaining blood samples were stored at −80°C on site at each hospital.

Molecular analysis was conducted at the University of Maryland School of Medicine, Baltimore, MD, USA. Parasite DNA was extracted from filter papers using the Qiagen DNA extraction kit (Qiagen, Valencia, CA, USA). *Plasmodium* species (*P. vivax*, *P. falciparum*, *Plasmodium ovale* and *Plasmodium malariae*) were detected by nested polymerase chain reaction (PCR) amplification of the small sub-unit ribosomal ribonucleic acid (ssrRNA) genes using the primers and thermal cycler conditions as described [29,30]. The amplified PCR products were visualized by 2–2.5% agarose gel electrophoresis, stained with ethidium bromide and visualized using the Bio-Rad gel doc system (Bio-Rad Laboratories, Hercules, CA, USA).

## Results

A total of 801 microscopy-positive samples was amplified by PCR along with positive and negative controls. By microscopy, 17% (n = 138) were identified as *P. falciparum*, 82% (n = 656) as *P. vivax* and 0.9% (n = 7) as mixed (Table 1). Nested PCR identified 18% (n = 128) as *P. falciparum*, 76% (n = 536) as *P. vivax*, 6% (n = 43) as mixed *P. falciparum/P. vivax*, and 94 samples as parasite-negative. *P. malariae* and *P. ovale* were not detected in any samples, and 27 of 30 microscopy-negative samples were parasite-positive by PCR.

**Table 1:** **Microscopic and PCR-based diagnosis of *Plasmodium *infections in clinical isolates**

**Species**	**Microscopy**	**PCR analysis**
*Plasmodium falciparum*	138 (17%)	128 (18%)
*Plasmodium vivax*	656 (82%)	536 (76%)
Mixed (*P. falciparum + P. vivax*)	7 (1%)	43 (6%)
*Plasmodium malariae*	0	0
*Plasmodium ovale*	0	0
Negative	0	94 (12%)

Discrepancies between PCR and microscopy were identified for 36 mixed-species samples by PCR (10 characterized as *P. falciparum* monoinfection and 26 as *P. vivax* monoinfection by microscopy) and 94 parasite-negative samples by PCR (characterized as *P. vivax* (82), *P. falciparum* (11) and mixed species infection (1) by microscopy). Slides were not available for re-examination in the case of discrepancies between microscopy and PCR, but PCR was repeated and results confirmed for all 130 samples.

Cohen’s kappa statistic to measure concordance between microscopy and PCR indicated that overall agreement for presence or absence of *Plasmodium* infection was high (kappa = 0.89), although agreement on mixed species infections was low (kappa = 0.49) due to the identification of a number of mixed species infections by PCR as either *P. vivax* or *P. falciparum* monoinfections by microscopy. Agreement on diagnosis as *P. falciparum* was high (kappa = 0.94) while agreement for *P. vivax* was slightly lower (kappa = 0.82), owing to the classification of 120 samples as *P. vivax*-positive by microscopy that did not have a detectable infection by PCR.

The age of subjects ranged from two months to 75 years, with a median of 24 years (Table 2). Age did not vary significantly by province. Overall, nearly two-thirds (64%) of the subjects were male. Males predominated in all study areas, ranging from 55% of subjects in Balochistan to 72% of subjects in Islamabad.

**Table 2:** Gender and age distributions of subjects by province

	**Males N (%)**	**Females N (%)**	**Age in years median (Range)**
**Balochistan**	52 (55%)	42 (45%)	29 (4 to 70)
**Islamabad**	62 (72%)	23 (28%)	26 (3 to 75)
**KPK**	231 (63%)	133 (37%)	22 (0.3 to 75)
**Punjab**	126 (64%)	71 (36%)	24 (0.4 to 65)
**Sindh**	38 (62%)	23 (38%)	28 (0.3 to 65)

Of the 801 microscopy-positive samples, 707 PCR-positive malaria cases were detected at all sites (Table 3). Khyber Pakhtunkhwa had the highest number of cases at 325, followed by Punjab with 157 cases. The overall prevalence of *Plasmodium* in Pakistan among treatment-seeking patients with suspected malaria was 6.6%: 5% *P. vivax*, 1.2% *P. falciparum* and 0.4% mixed *P.vivax/P. falciparum* infections. Balochistan, Sindh and Khyber Pakhtunkhwa provinces exhibited the highest prevalence of total *Plasmodium* infections, at 12.4, 11.1 and 10.8%, respectively (Table 3). Malaria prevalence in Punjab ranged from 1.7% in Lahore to 5.5% in Bhakkar cities. The capital city of Islamabad had an overall malaria prevalence of 4.6% (Table 3).

**Table 3:** **Prevalence (%) of *Plasmodium *infection among individuals presenting with symptoms consistent with malaria, by province and city**

			Prevalence*			
**Province**	**City**	**Suspected cases**	***P. vivax***	***P. falciparum***	**Mixed species**	**All species**
**Khyber Pakhtunkhwa**			**234 (7.7%)**	**65 (2.2%)**	**26 (1%)**	**325 (10.8%)**
	Bannu	895	7.7%	3.2%	1.1%	12.1%
	Hangu	445	6.7%	3.1%	1.1%	11.0%
	Peshawar	720	6.0%	0.4%	0.1%	6.5%
	Thall	950	9.7%	2.0%	1.1%	12.7%
**Punjab**			**114 (2.4%)**	**35 (0.7%)**	**8 (0.2%)**	**157 (3.3%)**
	Bhakkar	650	5.4%	0.2%	0.0%	5.5%
	Lahore	980	1.6%	0.0%	0.1%	1.7%
	Mainwali	1,205	2.6%	2.0%	0.3%	4.9%
	Muzaffarghar	688	1.2%	1.5%	0.4%	3.1%
	Rawalpindi	1,250	1.9%	0.0%	0.0%	1.9%
**Balochistan**			**49 (7.4%)**	**25 (3.8%)**	**8 (1.2%)**	**82(12.4%)**
	Quetta	280	1.1%	7.9%	1.4%	10.4%
	Zhob	380	12.1%	0.8%	1.1%	13.9%
**Sindh**			**58 (10.8%)**	**1 (0.2%)**	**1 (0.2%)**	**60 (11.1%)**
	Hyderabad	239	12.1%	0.0%	0.0%	12.1%
	Karachi	300	9.7%	0.3%	0.3%	10.3%
**Capital**			**81 (4.5%)**	**2 (0.1%)**	**0 (0.0%)**	**83 (4.6%)**
	Islamabad	1,800	4.5%	0.1%	0.0%	4.6%
**All**		**10,782**	**5.0%**	**1.2%**	**0.4%**	**6.6%**

*Prevalence was calculated by dividing the number of PCR-positive cases by the number of suspected cases.

*P. vivax* was the predominant malaria species in most regions of the survey. However, *P. falciparum* was found in the majority of *Plasmodium* infections in Quetta city of Balochistan province, and Muzaffarghar of Punjab province and in about one-third to one-half of all malaria infections in three other sites: Mainwali in Punjab, and Hangu and Bannu near Khyber Pakhtunkhwa’s Afghan border (Figure 1). Balochistan and Khyber Pakhtunkhwa provinces had the highest proportion of mixed *P*. *falciparum*/*P. vivax* infections, at 10 and 8%, respectively. *P. falciparum* malaria was virtually absent from the other three Punjabi sites, as well as from both cities in Sindh province and from Islamabad city (Table 4).

**Table 4:** **Number of samples PCR-positive for *Plasmodium *infection, by province and city**

		PCR positive samples*		
**Province**	**City**	***P. vivax***	***P. falciparum***	**Mixed species**
**Khyber Pakhtunkhwa**		**234 (72%)**	**65 (20%)**	**26 (8%)**
	Bannu	69	29	10
	Hangu	30	14	5
	Peshawar	43	3	1
	Thall	92	19	10
**Punjab**		**114 (73%)**	**35 (22%)**	**8 (5%)**
	Bhakkar	35	1	0
	Lahore	16	0	1
	Mainwali	31	24	4
	Muzaffarghar	8	10	3
	Rawalpindi	24	0	0
**Balochistan**		**49 (60%)**	**25 (30%)**	**8 (10%)**
	Quetta	3	22	4
	Zhob	46	3	4
**Sindh**		**58 (96%)**	**1 (2%)**	**1 (2)%**
	Hyderabad	29	0	0
	Karachi	29	1	1
**Capital**		**81 (98%)**	**2 (2%)**	**0%**
	Islamabad	81	2	0
**All**		**536 (76%)**	**128 (18%)**	**43 (6%)**

* Percentages indicate the proportion of PCR-positive samples for each species.

## Discussion

Malaria continues to be a serious public health problem in Pakistan. In 2008, 2.6 million malaria cases were reported nationwide with a mortality rate of 50,000 per year [13,31-33]. Over one million microscopy-confirmed malaria cases were reported from the Eastern Mediterranean region in 2010, 22% of which came from Pakistan [13]. It has been difficult to accurately estimate the prevalence of *Plasmodium* infection in Pakistan, largely because the diversity in prevalence and species distribution of malaria-causing parasites in different parts of the country has not been fully described.

This survey was conducted to provide up-to-date information on malaria prevalence throughout Pakistan. Because samples were collected from patients presenting with symptoms, one limitation is the potential for regional variation in treatment-seeking behaviour and access to treatment centres. Unfortunately, information on treatments taken prior to diagnosis was not collected and could not be included in this analysis. In addition, the timing of sample collection at each site may have coincided with varying levels of species-specific malaria transmission. All patients were sampled within the transmission season at each site, but during months that may have correlated with the presence of a particular species: *P. vivax* peaks between April and September while *P. falciparum* peaks between August and December [2,34-36]. Regional variations in peak times also occur, with a study in the hilly region of Balochistan reporting highest *P. vivax* infection and lowest *P. falciparum* infection in March and the opposite pattern in October [37]. These effects should be taken into account when assessing relatively small reported differences in the prevalence of malaria and in the proportion of *P. vivax* and *P. falciparum* cases among regions. Despite these limitations, the data presented here contribute to a more comprehensive and current characterization of malaria in Pakistan than has previously been available.

The predominance of malaria infection in males in their twenties has been observed in previous studies in this region [37,38]. Some hypotheses for the disproportionate number of male malaria patients include increased exposure because males are more likely to work outside and not be as well-covered as females, leading to an increased number of infected *Anopheles* bites, or that females may have diminished access to health care facilities and may not seek medical attention as frequently as males [26].

This survey indicated that the highest prevalence of malaria is found in Balochistan, followed by Sindh and Khyber Pakhtunkhwa, with lower prevalence in Punjab and the city of Islamabad. Similarly, the Malaria Case Management Desk Guide reported in 2007 that Sindh and Balochistan provinces contributed the majority of malaria cases in Pakistan, followed by Khyber Pakhtunkhwa province and the Federally Administered Tribal Areas, with the fewest cases reported in Punjab province [34]. This study also confirmed previous findings that *P. vivax* and *P. falciparum* are the two major *Plasmodium* species found in Pakistan, with *P. vivax* predominating in most regions [13,33,39].

In addition to the highest malaria prevalence in Pakistan, Balochistan also had the highest proportion of *P. falciparum* cases, due to the predominance of falciparum malaria in one of its districts, Quetta. Other studies have also reported a high prevalence of *P. falciparum* in this region, including in Quetta itself [39,40], and the cities of Zhob [37], East Balochistan [39], and Khuzdar in Balochistan province [41]. Although generally higher than in other regions of Pakistan, estimates of the proportion of *P. falciparum* infections within Balochistan province vary widely by location, from 15% in Zhob to nearly 90% in Quetta, indicating that geographic variation among districts is high.

The two sites in Sindh, Karachi and Hyderabad, had an overall malaria prevalence of 11%, with very few *P. falciparum* or mixed species infections. In contrast, previous studies have shown that approximately one-quarter to one-half of malaria cases were attributed to *P. falciparum* in these cities [42-44]. This difference may be attributable to the narrow sampling window in this province. The majority of samples was collected in April in Sindh, while previous observations indicate that peak infection of *P. falciparum* does not occur until later in the year [32].

Thall, Bannu and Hangu in Khyber Pakhtunkhwa province all showed high prevalence of malaria and a high proportion of cases attributed to *P. falciparum*. Other studies in this region have found a relatively high number of cases and varying levels of *P. falciparum*, from 16% of malaria cases in Buner [42] to 25% in Abbottabad and Bannu [33,45] to over 90% of cases in Manshera [46]. Cross-border migration may have contributed to the maintenance or surge of malaria in this region. Between 1979 and 1982, refugees from Afghanistan fled across the border into Khyber Pakhtunkhwa and Balochistan provinces [47]. This influx of a potentially more malaria-susceptible population may have overwhelmed the public health system [24], leading to greater disease. The movement of Afghan refugees into Baluchestan province, Iran, was estimated to result in a 24-36% increase in the number of malaria cases [48]. Recent internal displacement may also be contributing to the high prevalence of malaria. In 2009, inhabitants of several districts in the Federally Administered Tribal Areas and Khyber Pakhtunkhwa fled war conditions to settle in adjoining districts [49,50]. These large movements of vulnerable populations may have altered the distribution of malaria and malaria-susceptible people in the country [50], contributing to epidemics such as that reported in a Pakistani refugee camp in 2003 where incidence was as high as 100 cases per 1,000 persons per year [51].

Punjab province and the city of Islamabad had little malaria in comparison with other parts of the country. The prevalence of malaria in Islamabad has not previously been reported, but other studies in Punjab province reported similar results, with a microscopy-positive malaria prevalence of 2.4% in house-to-house surveys in 1999 [52] and low annual incidence, with less than one case per 10,000 per year between 2004 and 2009 [4]. Two contributors to the low level of malaria in Punjab have been suggested. Environmental changes resulting in waterlogging and salinization may have reduced suitable habitats for the more efficient vector, *Anopheles culicifacies*, while increasing breeding sites for the salt tolerant and less efficient vector, *Anopheles stephensi*[4]. Additionally, overall socio-economic conditions and the public health infrastructure are comparatively better in Punjab than in the rest of the country, and may play a role in more effectively controlling malaria [52].

Agreement between PCR and microscopy was high overall, but discrepancies were detected in samples that were microscopy-positive and PCR negative for *Plasmodium* and for samples that were *P. falciparum* or *P. vivax* monoinfections by microscopy but mixed species infections by PCR. Confirmation of PCR results in repeated assays suggests that the error likely lies in diagnosis by microscopy. Similar results have been reported in Pakistan [33] where nearly 15% of samples positive by microscopy were parasite-negative by PCR and in a 2007 study in Afghanistan, Iran, and Pakistan where 6.5, 2.2 and 23.5% of microscopically diagnosed *P. vivax* mono-infections, respectively, were mixed species by PCR [53]. The propensity to report febrile patients as parasite-positive has been reported [54], and factors influencing accuracy of microscopic diagnosis, including use of contaminated reusable microscope slides, staining artifacts, lack of microscopic expertise, and lack of quality assurance [55,56] have been well-documented.

It has been estimated that in countries where *P. vivax* is predominant, over one-third of patients presenting with acute *P. vivax* also harbour cryptic *P. falciparum*[57]. Misdiagnosis of mixed species infections can result in improper or incomplete treatment, particularly in countries like Pakistan, where chloroquine is used to treat *P. vivax* but is known to be ineffective against *P. falciparum.* Household surveys conducted in 2010 indicating that 80% of patients with suspected *P. falciparum* malaria (confirmed or unconfirmed) received chloroquine, 14% received an artemisinin-based combination therapy (ACT) and 16% were not treated with any anti-malarial [32]. Assessment of the quality of malaria microscopy in Pakistan may aid in identifying areas for improved diagnosis and treatment, particularly in regions endemic for both *Plasmodium* species.

## Conclusions

Pakistan faces several challenges in the management and control of malaria, including misdiagnosis, lack of diagnostic facilities, use of presumptive treatments and unavailability of ACT [58-60]. Information on the burden and species distribution of malaria is critical for guiding national and provincial efforts in diagnosis and effective treatment. The results of this study indicate that malaria control efforts should be focused on Khyber-Pakhtunkhwa, Balochistan and Sindh provinces, where malaria prevalence is highest, with an emphasis on improving species diagnosis and ACT availability for treatment of *P. falciparum* in Quetta city of Balochistan, parts of Khyber Pakhtunkhwa, and Punjab province.

## Competing interests

The authors declare that they have no competing interests.

## Authors’ contributions

AAK designed the study, carried out the laboratory experiments, and conducted data analysis. AAK and MV drafted the manuscript. MFN, HSS and AY participated in sample and data collection and microscopy. KS aided in laboratory experimental design and execution. MV, LK, SAM and CVP provided guidance and coordination for study design laboratory analyses and data analysis, and edited and revised the manuscript. All authors read and approved the final manuscript.
